# Editorial: Origin of Tropical Diversity: From Clades to Communities

**DOI:** 10.3389/fgene.2016.00186

**Published:** 2016-10-20

**Authors:** James E. Richardson, R. Toby Pennington

**Affiliations:** ^1^Programa de Biología, Universidad del RosarioBogotá, Colombia; ^2^Tropical Diversity Section, Royal Botanic Garden EdinburghEdinburgh, Scotland

**Keywords:** tropical, diversity, phylogeny, phylogeography, ecology, paleontology, community

For centuries, one of the main questions in relation to the Earth's biological diversity has been disentangling the causes for the comparatively high diversity in tropical zones, particularly the Neotropics. This volume aimed to demonstrate how molecular phylogenetics, phylogeography, paleontology, and paleoecology contribute to our understanding of the processes that gave rise diversity in the tropics.

The response of authors to our initial invitation to participate was overwhelming, and the resulting manuscripts are brought together in this eBook. We have attempted to order the contributions, placing first those that deal with general conceptual or methodological issues and that are relevant to all parts of the tropics. These included studies on how tropical diversity was generated and/or is maintained at those local scales (Cannon and Lerdau; Beheregaray et al.; Collevatti et al.). The next group of articles focuses on specific taxa, though in most cases these are distributed throughout the tropics, and so address global questions. The final group of articles addresses studies confined to single major tropical regions including Africa and Australia.

## General and conceptual contributions

The first group of general and conceptual papers leads with Cannon and Lerdau who presented a study that suggested that inter-specific gene flow may play an important role in preventing extinction, particularly of rare species, and thus maintaining species diversity. Although the number of well corroborated examples of hybridization amongst tropical plant species is increasing (examples include Duminil et al., 2012), assessing the generality of Cannon and Lerdau's suggestion will require many more studies of the frequency of interspecific crosses in tropical floras. Beheregaray et al. reviewed studies that have demonstrated ecological speciation in the tropics and encourage an approach using a conceptual and analytical framework based on genetic data and simulation modeling. Collevatti et al. presented statistical and macroecological approaches in a multi-model inference framework that can be applied to comparative phylogeography. The method employs direct reconstruction of lineage divergence in time and space together with ecological niche modeling and coalescent simulation. This multi-model approach is suggested to be more likely to determine correctly the micro-evolutionary processes and spatial context of lineage divergence.

General explanations for the latitudinal gradient in species richness were postulated by Hurlbert and Stegen and through empirical studies (e.g., Richardson et al.; Antonelli et al.). Hurlbert and Stegen highlighted the deficiencies in evaluating the predictions of a single diversity hypothesis that does not rule out alternatives. Richardson et al. and Antonelli et al. both indicated that tropical lineages of plants had not evolved at a faster rate than temperate ones, and supported the hypothesis that the tropics are more species rich because tropical conditions occupied greater areas and have existed for longer periods than temperate ones. Antonelli et al. analyzed c. 22,600 species and c. 20 million geo-referenced records of angiosperms and suggested that differential rates of diversification were not responsible for the latitudinal gradient as there were no significant differences between speciation and extinction in tropical and non-tropical lineages. However, Pyron and Wiens (2013) showed that in amphibians the latitudinal gradient could be explained by a combination of higher speciation in the tropics, greater extinction in temperate zones, and low dispersal out of the tropics compared with an alternative explanation of colonization of the tropics from temperate regions.

## Taxon based contributions

Although at global scales diversification rates of plants between tropical and temperate regions do not appear to differ in the studies of Antonelli et al. and Richardson et al., care must be taken in accounting for regional differences in diversification rates. Richardson et al. indicated a more rapid diversification for the neotropical genus *Theobroma* and allies than in related lineages from other tropical and temperate regions. The diversification of *Theobroma* coincided with, and may have been driven by, Andean uplift, supporting the ideas of Gentry (1982) that the greater diversification in the Neotropics might have resulted from the greater and more recent orogenic events there in comparison with Africa or Asia.

In this research topic, we aimed to emphasize the whole tropics because a global synthesis is necessary to place varying local patterns and processes in context. This global perspective was covered in a review of patterns in diversity in tropical reef fishes (Cowman et al.), diversification processes in climbing palms (Couvreur et al.), and in tropical trees (Armstrong et al.; Weeks et al.). Armstrong et al. indicated that Neotropical lineages of the predominantly lowland tropical forest genus *Manilkara* (*Sapotaceae*) had a faster diversification rate than African or Asian ones. However, comparisons of the capacity of closely related families to evolve into distinct biomes are also necessary. Weeks et al. compared climatic niche evolution in two broadly distributed sister families. In Anacardiaceae, adaptation to different climates seems to have played a prominent role in diversification, while in Burseraceae much less so. Couvreur et al. demonstrated how the morphological changes necessary to evolve from non-climbing to climbing habit may have played an important role in palm diversification leading to the recent speciation of one-fifth of extant palm species.

## Regional contributions

The Neotropics perhaps remain the major tropical region that is best studied in the field of evolutionary diversification. In this volume, Antonelli et al. demonstrated that the American tropics are the area with the highest evolutionary turnover and rate of emigration in comparison to other major tropical regions. Carrillo et al. highlighted that, for mammals, the Great American Biotic Interchange between the Laurasian and Gondwanan land masses of the Americas began c. 7–10 mya and that the first migrations involved temperate taxa, although this result may have been skewed by greater sampling at higher latitudes. Patterns of migration between Laurasian Central America and Gondwanan South America were also assessed in Malpighiacaeae (Willis et al.) indicating that a remarkable amount of dispersal from South to Central America contributed more to increasing phylogenetic diversity there than *in situ* diversification.

Based on paleoecological evidence, it was thought that Quaternary glacial cycles fueled neotropical diversification through the alternation of glacial aridity and warmer, wetter inter-glacials that led to contraction and expansion of species ranges (e.g., Haffer, 1969). The universality of “refuge theory” was subsequently challenged by data that suggested that many extant species were in fact of pre-Pleistocene origin (e.g., reviewed by Moritz et al., 2000). This debate has primarily been argued in studies of Neotropical lineages but processes may have been different in other tropical regions. In this collection of papers, Damasceno et al. assessed the neglected vanishing refuge model (VRM), first described by Vanzolini and Williams (1981), which describes a process of diversification that implicates both vicariance and divergent selection. For the neotropical lizard species, *Coleodactylus meridionalis*, Damasceno et al. found that environmental and genetic analyses fitted the predictions of the VRM, but physiological data did not. Based on fossil data, Collins et al. demonstrated species persistence in the Galápagos highlands in mesic micro-refugia during the last glacial maximum. It was suggested that these micro-refugia may be areas of preservation and resilience to increasing aridity as global temperatures increase.

In order to make better comparisons amongst tropical regions, more studies are needed that focus on Africa, the Indian sub-continent and Australasia, all of which appear to be more neglected in the literature of tropical diversification and biogeography. New studies may provide insights of the effects of differing regional climatic and geological histories. For example, the scale of Pleistocene aridification in Africa was much greater than in the Neotropics or Southeast Asia. Diversification in Southeast Asia may have proceeded differently as a result of the mostly archipelagic nature of the region, its complex tectonic history, and Quaternary sea-level changes (Richardson et al., 2014).

This ebook includes extensive reviews and novel studies on Afro-Madagascan taxa including a review of the evolution of African plant diversity (Linder) and the Rand flora (Pokorny et al.), which do much to explain patterns of diversity within Africa and the comparative paucity of species on that continent compared to other parts of the tropics. Linder implicated a role for phylogenetic niche conservatism in the construction of different elements of the African flora. The austro-temperate flora has recruited lineages from similar southern hemisphere temperate floras and the lower latitude floras from those of the tropical regions of other continents. The poverty of the African tropical flora may have been related to the spread of C4 grasslands and the change to fire-regulated ecologies. Pokorny et al. revealed how different elements of the Rand flora, which occupies the continental margins of Africa and adjacent islands, have adapted to differing levels of intensity of aridification at different spatial and temporal scales. The age of disjunctions coincide with the climatic affinities of each Rand Flora lineage. Wollenberg Valero emphasized the importance of both topographic and riverine barriers in generating genetic divergence along with body size changes in lineage diversification in microendemic species of Madagascan leaf litter frogs. Ley et al. studied the possible impacts of Pleistocene aridification on phylogeographic patterns in eight species of herbs in the family Marantaceae. They suggested that areas of high diversity might have been both Quaternary refuges and/or secondary contact zones. These patterns in herbaceous plants were similar to those found among tropical tree species (Dauby et al., 2014), especially in the study area of southern Lower Guinea.

In a volume that we edited on the historical assembly of major biomes in 2004 (Pennington et al., 2004), which had a major focus on the tropics, the topics of phylogenetic diversity and especially community phylogenetics were hardly mentioned because they were in their infancy. In the subsequent decade, they have become influential, and two contributions to this volume reflect this. The value of mega-phylogenies generated from species found in multiple forest plots was assessed by Erickson et al. Phylogenetic resolution and estimates of phylogenetic diversity were better and more consistent in the mega-phylogeny than in phylogenies of individual plots. Based on specimen records and a generic phylogeny of ferns, Nagalingum et al. identified a hotspot of both taxic and phylogenetic diversity in the wet tropics of northeastern Australia. The variable that best explained phylogenetic diversity was annual precipitation, but the species-energy hypothesis was supported with the correlation of phylogenetic diversity to precipitation plus radiation. Northeastern Australia has also been shown to be a zone in which elements from Laurasia have recently mixed with Australian (or Gondwanan) ones that could potentially result in high phylogenetic diversity (Richardson et al., 2012).

It is increasingly clear from studies presented in this volume and previous ones (e.g., McKenna and Farrell, 2006; Moreau and Bell, 2013) that the timing and drivers of tropical diversification are a result of multiple factors acting at different spatial and temporal scales and in different ways in different taxonomic groups. This volume contributes a substantial number of studies from an empirical perspective and also presents novel ways of using new and traditional forms of data. Over the past quarter of a century huge progress has been made in developing our understanding of the events that gave rise to the diversity of tropical regions. The potential for furthering our understanding of these processes has been boosted by new DNA sequencing technologies that permit the accumulation of billions of data points that will allow us to reconstruct more accurately the evolutionary history of tropical organisms. A continuing challenge will be the gathering of more samples, particularly from lesser studied tropical regions, which will allow regional biota to be placed in a global context. Our aim was to include studies from some of those more neglected areas, but more are still needed, particularly in Africa and Asia. We hope that this volume will serve to encourage further studies.

## Author contributions

All authors listed, have made substantial, direct and intellectual contribution to the work, and approved it for publication.

### Conflict of interest statement

The authors declare that the research was conducted in the absence of any commercial or financial relationships that could be construed as a potential conflict of interest.
